# E2F1 binds to the peptide-binding groove within the BIR3 domain of cIAP1 and requires cIAP1 for chromatin binding

**DOI:** 10.1371/journal.pone.0206253

**Published:** 2018-10-25

**Authors:** Jennifer Allègre, Jessy Cartier, Valérie Glorian, Nathalie Droin, Baptiste Dumetier, Cémile Kayaci, Jean Berthelet, Simon Gemble, Céline Vuillier, Laurent Maillet, Carmen Garrido, Laurence Dubrez

**Affiliations:** 1 Institut National de la Santé et de la Recherche Médicale (Inserm), LNC UMR1231, Dijon, France; 2 Université de Bourgogne Franche-Comté, LNC UMR1231, Dijon, France; 3 Inserm U1170, Gustave Roussy, Villejuif, France; 4 CRCINA, INSERM, U1232, Université de Nantes, Nantes, France; H. Lee Moffitt Cancer Center & Research Institute, UNITED STATES

## Abstract

The cellular inhibitor of apoptosis 1 (cIAP1) is an E3-ubiquitin ligase that regulates cell signaling pathways involved in fundamental cellular processes including cell death, cell proliferation, cell differentiation and inflammation. It recruits ubiquitination substrates thanks to the presence of three baculoviral IAP repeat (BIR) domains at its N-terminal extremity. We previously demonstrated that cIAP1 promoted the ubiquitination of the E2 factor 1 (E2F1) transcription factor. Moreover, we showed that cIAP1 was required for E2F1 stabilization during the S phase of cell cycle and in response to DNA damage. Here, we report that E2F1 binds within the cIAP1 BIR3 domain. The BIR3 contains a surface hydrophobic groove that specifically anchors a conserved IAP binding motif (IBM) found in a number of intracellular proteins including Smac. The Smac N-7 peptide that includes the IBM, as well as a Smac mimetic, competed with E2F1 for interaction with cIAP1 demonstrating the importance of the BIR surface hydrophobic groove. We demonstrated that the first alpha-helix of BIR3 was required for E2F1 binding, as well as for the binding of Smac and Smac mimetics. Overexpression of cIAP1 modified the ubiquitination profile of E2F1, increasing the ratio of E2F1 conjugated with K11- and K63-linked ubiquitin chains, and decreasing the proportion of E2F1 modified by K48-linked ubiquitin chains. ChIP-seq analysis demonstrated that cIAP1 was required for the recruitment of E2F1 onto chromatin. Lastly, we identified an E2F-binding site on the cIAP1-encoding birc2 gene promoter, suggesting a retro-control regulation loop.

## Introduction

Inhibitors of Apoptosis (IAPs) are a family of cell signaling regulators involved in a large panel of fundamental cellular processes including cell death, cell proliferation, cell differentiation, innate immunity and inflammatory responses [1]. Belonging to this family, cIAP1 is a really interesting new gene (RING)-containing E3-ubiquitine ligase. E3-ligases carry out the final step of the ubiquitination cascade aiming to covalently conjugate single ubiquitin molecules or polyubiquitin chains to a substrate protein. This post-translational modification is a very efficient and fast mechanism of controlling the fate of intracellular proteins. It brings the plasticity required for the cell to quickly adapt to changing intracellular or environmental conditions. Depending on the nature of the conjugated ubiquitin chain, it can modify the stability, activity, or subcellular location of protein substrates, or their recruitment to signaling platforms. RING-containing E3-ligases catalyze the transfer of ubiquitin from an E2-congating enzyme to a lysine amino-acid residue of client proteins [2]. E3s are responsible for substrate specificity. cIAP1 recognizes and binds specific protein partners thanks to the presence of three baculoviral IAP repeat domains (BIRs) located at the N-terminal extremity [3]. BIRs consist of 3 short β-strands and 4–5 α-helices structured by a single zinc ion coordinated by three cysteines and one histidine residue. Although they are conserved, variable surface-exposed residues confer binding specificity on each BIR [4]. BIR1 is responsible for binding the tumor necrosis factor receptor-associated factor 2 (TRAF2). BIR2 and BIR3 each display a hydrophobic surface groove formed by the β1–3 strands and the α3-helix. This allows the anchorage of a conserved tetra-peptide motif called the IAP binding motif (IBM). IBMs have been found in a number of intracellular proteins, most of which are mitochondrial [5], such as the second mitochondrial-derived activator of caspase/direct inhibitor of apoptosis-binding protein with low pI (Smac/Diablo)[6, 7], the high-temperature requirement A2 (HtrA2)[8], the PGAM family member 5, mitochondrial serine/threonine protein phosphatase (PGAM5) [9] for which IBM is exposed after removal of the N-terminal mitochondrial targeting motif during proteolytic maturation of the protein. The IBM of non-mitochondrial proteins is located just downstream of the initiating methionine, for example in the cases of NF-kappa-B (NF-κB)-inducing *kinase* (NIK) and the checkpoint kinase 1 (chk1) [10, 11]. Alternatively, it may be exposed at the N-terminal extremity of the cleavage product upon caspase- or calpain-mediated processing during apoptosis or reticular stress such as in the cases of caspase 3, 7 or 9 or the eRF3/GSTP releasing factor [12–14]. Alternative modes of interaction with BIR3 also exist, involving unconventional IBMs [15] or attachment to another BIR-binding interface [16].

Most of the identified cIAP1 partners are involved in the tumor necrosis factor receptor (TNFR) and/or NF-κB activating signaling pathways. Thus, cIAP1 appears to be an extremely potent regulator of the innate immune response and inflammation [17]. cIAP1 can also bind apoptosis signaling pathway components such as the XIAP antagonist Smac and caspases-3, 7 and 9 [13, 18–20]. We, and others, have identified novel cIAP1 partners that enlarge the spectrum of activity. These include GTPases of the Rho Family that control actin cytoskeleton reorganization [21, 22], the c-RAF serine/threonine kinase [23], the heat-shock protein 90β (HSP90β)[24], chk1 [11], the transcription co-factors c-myc antagonist Max-dimerization protein-1 (Mad1)[25], the vestigial-like 4 (Vgl-4)[26], the transcription factors C/EBP homologous protein (CHOP)[27] and the E2 promoter binding factor 1 (E2F1)[28].

In previous work, we demonstrated that E2F1 was a ubiquitination substrate of cIAP1 [29]. In cells that express a nuclear cIAP1, this interacts with E2F1 in all phases of the cell cycle. We showed that cIAP1 was recruited to CCNE and CCNA gene promoters in the S phase of the cell cycle along with the transcription factor and that this stimulated E2F1 transcriptional activity [28]. We found that cIAP1 was required for the stabilization of E2F1 in response to DNA damage or during the S phase of the cell cycle [29]. The present study aims to explore the cIAP1-E2F1 interaction. We demonstrate that E2F1 binds the BIR3 domain of cIAP1 in a peptide-binding groove-dependent manner and that the first alpha helix of BIR3 is required for the interaction. Overexpression of cIAP1 modifies the ubiquitination profile of E2F1, increasing the K11 and K63-ubiquitin-conjugated forms and decreasing K48-ubiquitinated E2F1. A ChIP-seq analysis demonstrates that cIAP1 is required for the general recruitment of the transcription factor to DNA. Finally, we show that cIAP1-encoding *BIRC2* is an E2F1 target gene. This suggests the presence of a retro-control regulation loop.

## Materials and methods

### Plasmid constructs

The DNA constructs used were: pGEX-4T1, pGEX-cIAP1^wt^, pGEX-cIAP1^L47A^, pGEX-cIAP1^BIR1-UBA^ (amino-acids 1–483), pGEX-cIAP1^CARD-RING^ (amino-acids 452–618), pGEX–cIAP1^BIR1-2^ (amino-acids 1–258), pGEX-cIAP1^BIR2-3^ (amino-acids 181–363), pGEX-cIAP1^BIR1^ (amino-acids 34–129), pGEX-cIAP1^BIR2^ (amino-acids 170–260), pGEX-cIAP1^BIR3^ (amino-acids 256–358) [30]; pCMV-3HA-E2F1; pCI, pCI-cIAP1, pCI-cIAP1^F616A^; pCMV-βGal, pGL3 human CCNE promoter [28, 29]; pcDNA3.1-6His-Ub wt, 6His-Ub-K11only, 6His-Ub-K48only and 6His-Ub-K63only mutants in which all K residues except for K11, K48 or K63 were changed into R; pcDNA3-myc-skp2 (Addgene plasmid #19947, Addgene, Cambridge, UK)[31] and pCS2-HA-cdh1 (addgene plasmid #11596)[32].

The BIR3 deletion mutant A-H DNA sequences were amplified from the BIR3 DNA by PCR and cloned into pGEX-4T1 (GE Healthcare Life Sciences, Velizy Villacoublay, France). For cIAP1^ΔBIR3^ (deletion of amino acids 271–357) and cIAP1^Δα1BIR3^ (deletion of amino acids 281–294) constructs, pCI-cIAP1 and pGEX-cIAP1 were amplified using specific primers encompassing the BIR3 (amino acids 271–357) or the BIR3-α1-helix (amino-acids 281–294) (ΔBIR3 primers sense: 5’-CGGTCGACCAGCTGTTGTCAACTTCAGATACC-3’, antisense:5’-CGGTCGACTGCATGTGCTGCATGCTCAG-3’; Δα1BIR3 primers sense: 5’-CGGTCGACCCATCTAGTGTTCCAGTTCAGCC-3’, antisense: 5’-CGGTCGACCATGCTCAGATTTGAAATGCTAAACCTC-3’) and containing a Sal1 restriction site. Plasmids were then closed using T4 DNA-ligase (Promega, Charbonnières-les-bains, France). The human birc2 promoter region that contains the first exon, 2 putative E2F binding sites and 2 NF-κB binding sites (982 pb) was synthesized and cloned into pGL3 between the SacI and HindIII sites (construction subcontracted to Eurogentec SA, Seraing, Belgium).

### Cell culture and treatment, treatment and transient cell transfection

A human Cervical Carcinoma Cell line (HeLa, ATCC CCL-2) and human Osteosarcoma epithelial cell line (U-2 0S, ATCC HTB-96) were grown in DMEM (Dulbecco’s modified Eagle‘s medium; Dominique Dutscher, Brumath, France) that contained 10% fetal bovine serum (FBS, Dominique Dutscher), at 37°C in a 5% CO2 atmosphere and 95% humidity. Cells were transiently transfected using JetPEI (Polyplus transfection kit, Ozyme, France). Lipofectamine RNAimax reagent (Fischer Scientific) was used to transfect siRNAs. cIAP1 si-RNA oligonucleotides were designed and purchased from Qiagen S.A.S. (Courtaboeuf, France). Cells were treated with 17nM GDC-0152 or 1μM Birinapant (Selleckmed, Euromedex, Souffelweyersheim, France) for 1hour, with cycloheximide (CHX) 100μg/mL (Sigma-Aldrich, St Quentin Fallavier, France) for up to 16 hours. MG132 (Sigma-Aldrich) was used at 1μM or 5μM overnight. Cells were synchronized using a 2 mM thymidine (Sigma-Aldrich) double block.

### Glutathione S-transferase (GST) pull-down assay

GST-pull-down analysis was performed as described previously [21]. The E2F1 recombinant protein was produced from pCMV-E2F1 using the TnT Quick Coupled Transcription/Translation System (Promega) following the manufacturer’s instructions. The Smac-N7 peptide (Calbiochem, Merck, Molsheim, France), the Smac mimetic BV6 (Selleckmed) or DMSO (vehicle buffer) was added to GST-fusion proteins 30 min. before the cell lysate.

### Cell extracts, immunoprecipitation and immunoblotting

Cells were lysed in RIPA Buffer (NaCl 150 mM, NP40 1%, Sodium Deoxycholate 0.5%, SDS 0.1%, Tris pH7.5 50 mM and protease inhibitor cocktail). For immunoprecipitation, cells were lysed in IP lysis buffer (150 mM NaCl, 50mM Tris HCl pH 7.4, 20 mM EDTA, 0.5% NP40, 1mM DTT, 5mM N-Ethylmaleimide; Sigma-Aldrich) and protease inhibitors for 30 min at 4°C. Lysates were pre-cleared using 20μl of Protein A/G+ Agarose beads (Sigma-Aldrich), and incubated overnight at 4°C with mouse IgG1 purified anti-HA.11 (Biolegend, Ozyme, Montigny-le-Bretonneux, France), goat polyclonal anti-cIAP1 antibody (R&D systems Europe, Lille, France) or mouse or goat IgG. Beads were then washed in IP lysis buffer and denatured in Laemmli buffer before immunoblot analysis. Proteins were separated by SDS-PAGE and electro-transferred onto polyvinylidene difluoride membranes (GE Healthcare). Blots were probed with the following antibodies: rabbit polyclonal anti-E2F1 (C20, Santa Cruz, Biotechnology, Clinisciences, Nanterre, France), goat polyclonal anti-GST (Rockland, Tebu-bio SAS, Le Perray en Yvelines, France), rabbit polyclonal anti-TRAF2 (Stressgen, Enzo Life Sciences, Villeurbanne, France), mouse monoclonal anti-HA.11 (Biolegend), mouse monoclonal anti-HSC70 (Santa Cruz Biotechnology), goat polyclonal anti-cIAP1 (AF8181, R&D systems Europe), mouse anti-Smac/DIABLO (BD Transduction Laboratories, BD Biosciences, Le Pont de Claix, France), mouse anti-Myc (Cell Signaling technology, Saint Quentin Yvelines, France), mouse anti-β-actin (AL978) and mouse anti-α-tubulin (clone AA13) (Sigma-Aldrich), monoclonal FK2-HRP-conjugated anti-ubiquitin (Enzo Life Sciences, Villeurbanne, France). Detection was performed using peroxidase-conjugated secondary antibodies and a chemiluminescence detection kit (Clarity Western ECL substrate, Bio-Rad, Marnes-la-Coquette, France). The signal intensity was quantified using Image J software.

### Gene reporter assay

The transactivation activity of E2F1 was analyzed as previously described [28].

### Ubiquitinylation assay

HeLa cells were transfected with 5μg of each plasmid encoding wt ubiquitin, K11only, K48only or K63only mutants, HA- wt E2F1 and cIAP1 wt or mutants. Cells were treated overnight with 1μM MG132 (Sigma-Aldrich) and with 10 μM of the non-selective DUB inhibitor PR619 (LifeSensors, Tebu-bio SAS, France) before cell lysis. 3HA-E2F1 was immunoprecipitated with anti-HA antibody, and the ubiquitination of E2F1 was revealed using monoclonal FK2-HRP-conjugated anti-ubiquitin (Enzo Life Sciences).

### Chromatin immunoprecipitation assay

Protein/DNA complexes were cross-linked with 1% formaldehyde. Chromatid was isolated and sonicated. Ten percent of the lysate containing digested chromatid was conserved for input. The remaining lysate was diluted and immunoprecipitated with 5μg of rabbit anti-E2F1 (C-20) or rabbit control Igg (Santa Cruz biotechnology) overnight. Elution and DNA recovery were carried out according to recommendations of PIERCE Agarose Chip Kit (Thermo Scientific). Real-time PCR was performed as previously described [28] using primers flanking the E2F-binding site in *birc2* promoters (BS1 primer sense: (5’-TGAGGTGACACAGGGTAGGA-3’, antisense: 5’- GGTTTCCCAAAACTCAAACG-3’; BS2 primer sense: 5’- ACTCTTCTGGCCCTTGGACT -3’, antisense: 5’-AAACTTAGCCCTCGGCGTT -3’). For the ChIP-seq analysis, enriched DNA from ChIP and input DNA fragments were end-repaired, extended with an ‘A’ base on the 3′-end, ligated with indexed paired-end adaptors (NEXTflex, Bio Scientific, Saint-Marcel, France) using the Bravo Platform (Agilent), size-selected after 4 cycles of PCR with AMPure XP beads (Beckman Coulter, Villepinte, France) and amplified by PCR for 10 more cycles. Library quality was assessed by a bioanalyzer using a High Sensitivity chip.

## Results

### E2F1 binds to the BIR3 domain of cIAP1 in a peptide-binding groove-dependent manner

**Fig 1:**
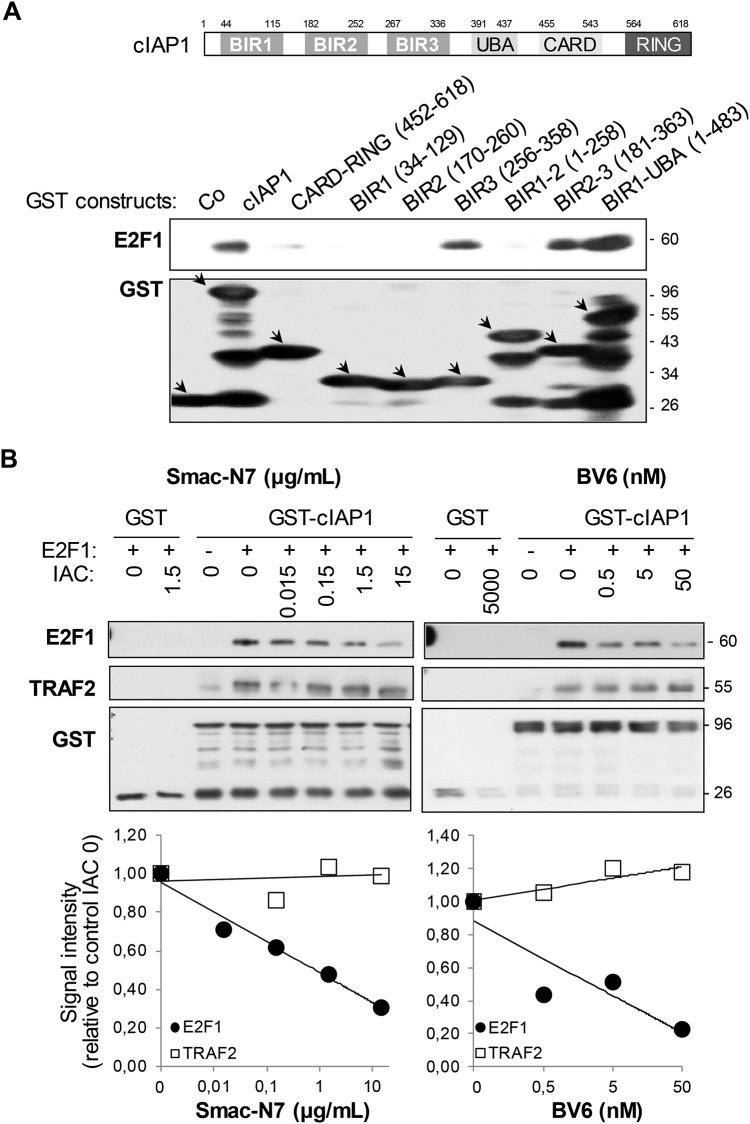
E2F1 interacts with the BIR3 peptide-binding groove. GST-pull-down analysis of the interaction of E2F1 with cIAP1. GST-fusion proteins were produced in bacteria, immobilized on glutathione-sepharose and incubated in the presence of E2F1 recombinant protein (A) or HeLa cell lysate (B). The interaction was revealed by Western-blot analysis. Increasing concentrations of the Smac-N7 peptide (μg/mL) or the Smac mimetic BV6 (nM) or DMSO vehicle buffer corresponding to the higher concentration (0) were added to immobilized GST-fusion proteins 30 min before the interaction step (B). E2F1 and TRAF2 binding were quantified using Image J software and related to binding without IAC (lower panel). One representative experiment is shown.

We previously demonstrated an interaction of cIAP1 with E2F1 [28]. We used a GST-pull-down assay to map the cIAP1 region required for this interaction. The full-length cIAP1 and all the cIAP1 deletion mutants containing the BIR3 domain (isolated BIR3, BIR 2–3 and BIR1-UBA constructs) interacted with E2F1, whereas the BIR3-less mutants (BIR1, BIR2, BIR1-2, CARD-RING) did not or only did so weakly (Fig 1A). BIR3 contains a surface hydrophobic groove that specifically binds the IBM exposed at the N-terminal extremity of several IAP partners (S1 Fig). The Smac-N7 peptide corresponding to the first 7 amino-acids of Smac encompassing the IBM or the Smac-mimetic BV6 competed with E2F1 for cIAP1 interaction in a dose-dependent manner (Fig 1B) demonstrating that E2F1 used the peptide-binding groove to bind cIAP1. In comparison, cIAP1-TRAF2 binding that does not involve the BIR-peptide-binding groove was not affected (Fig 1B).

We then looked for a potential IBM in the E2F1 primary protein structure. The IBM-BIR peptide-binding groove interacting mode has been extensively studied. This led to the establishment of an IBM consensus sequence A(K/T/V)(P/A/E)(F/E/I/S/Y) [3,4] with some possible variation (Panel A in S1 Fig). Such IBMs require exposure at the very N-terminus of the protein to anchor into the peptide-binding groove. The IBM is located just downstream of the initiating methionine [10, 11] or is exposed at the N-terminal extremity following post-translational cleavage [12–14]. cIAP1 interacts with the full-length-, 60 kDa-E2F1 (Fig 1A and 1B). Therefore, we postulated that the sequence downstream of the initiating methionine that starts with an alanine residue could be a putative IBM. We used an E2F1 mutant in which the two first amino acids (MA) had been substituted by a 3xHA tag to mask the potential IBM. We found that cIAP1 could still bind the 3HA-conjugated E2F1 in co-precipitation experiments, suggesting the presence of an alternative mode of interaction (Panel B in S1 Fig).

### The BIR3 α1-helix is required for E2F1 binding and an IBM-dependent interaction

**Fig 2:**
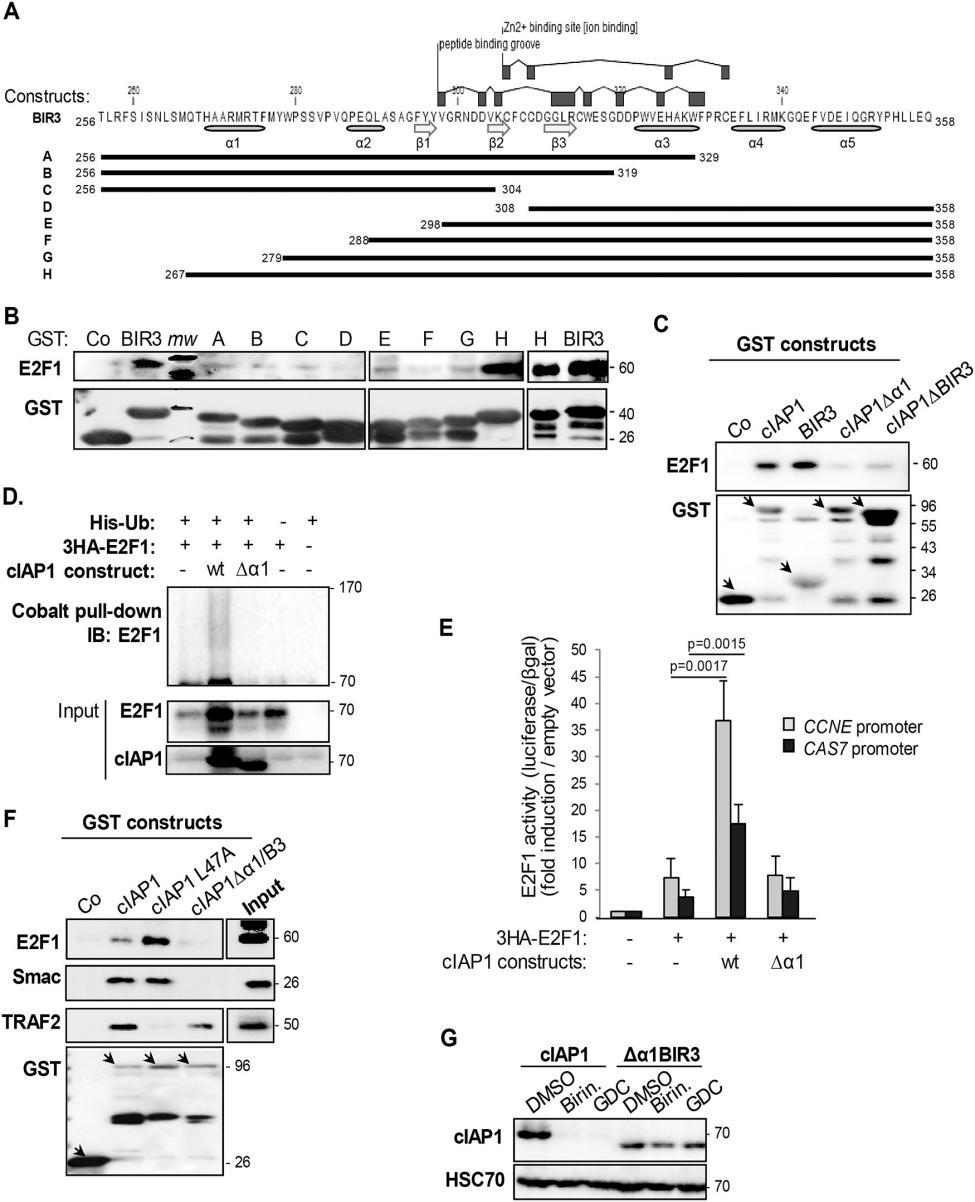
The first α-helix is required for the E2F1 interaction. (A) Primary structure of the cIAP1-BIR3 and BIR3 A-H deletion mutants. (B & C) GST-pull-down analysis of the interaction of E2F1 with the BIR3 deletion mutants described in A (B) or with wt cIAP1, isolated BIR3 or cIAP1 devoid of the BIR3 α1-helix (cIAP1Δα1) or the whole BIR3 (cIAP1ΔBIR3)(C). GST-fusion proteins were produced in bacteria, immobilized on glutathione-sepharose and incubated in the presence of E2F1 recombinant protein (B) or HeLa cell lysate (C). The interaction was revealed by Western-blot analysis. Mw: molecular weights. (D) Analysis of ubiquitination of E2F1 in HeLa cells transfected with wt 3HA-E2F1, His-Ubiquitin, an empty vector or a vector encoding wt cIAP1 or the BIR3-α1-helix deleted mutant. Ubiquitinated proteins were pulled-down using cobalt beads, and ubiquitinated E2F1 was revealed using an anti-E2F1 antibody. (E) E2F1 transcriptional activity was assessed in luciferase gene experiments using constructs in which luciferase expression was controlled by the *CCNE* or *CAS7* promoter. Luciferase activity was normalized to β-galactosidase activity and expressed as fold-induction of promoter activity measured in cells transfected with empty vectors. The mean ± S.D. of at least 3 independent experiments is shown. Statistical analysis was performed using Student’s *t* test. (F) GST-pull-down analysis of the interaction of E2F1, Smac or TRAF2 with wt cIAP1, cIAP1 devoid of the BIR3 α1-helix (cIAP1Δα1) or cIAP1 altered in the TRAF2 interacting site (cIAP1-L47A). GST-fusion proteins were produced in bacteria, immobilized on glutathione-sepharose and incubated in the presence of HeLa cell lysate. The interaction was revealed by Western-blot analysis. (G) Western blot analysis of cIAP1 in HeLa cells expressing wt cIAP1 or cIAP1Δα1, and treated for 1 hour with 1μM Birinapant (Birin.) or 17 nM GDC-0152 (GDC). HSC70 was used as a loading control. One representative experiment is shown.

To deal in depth with interaction mechanisms, we constructed a series of truncated cIAP1-BIR3 mutants (Fig 2A). As expected, altering the hydrophobic surface groove or the Zn^2+^ binding site (amino acids 298 to 334) drastically compromised E2F1 binding (Fig 2B, constructs A-E). Interestingly, although the peptide-binding groove is not touched, the deletion of amino acids 267 to 279 containing the first α helix was enough to disturb BIR3-E2F1 binding (Fig 2B, construct G compared to construct H). The deletion of the BIR3 α1-helix (cIAP1 Δα1B3 construct) as well as the deletion of the entire BIR3 (ΔBIR3) altered the capacity of cIAP1 to bind E2F1 (Fig 2C). This confirmed the importance of these regions for the cIAP1-E2F1 interaction. As was previously demonstrated [28, 29], cIAP1 increased the expression of E2F1 in a poly-ubiquitinated form (Fig 2D) that is associated with an enhanced transcriptional activity on the CCNE and CAS7 promoters (Fig 2E). Deletion of the BIR3 α1-helix also completely abolished these properties (Fig 2D and 2E).

The deletion of the BIR3 α1-helix also abolished the capacity of cIAP1 to interact with the IBM-containing Smac, but did not compromise the cIAP1–TRAF2 interaction (Fig 2F), which is inhibited by the substitution of the leucine 47 residue into alanine (cIAP1 L47A construct) [21, 28, 30]. Smac-mimetic compounds such as Birinapant or GDC-0152 target the BIR peptide-binding groove of cIAP1 that results in cIAP1 auto-ubiquitination and its proteasome system-mediated degradation [13]. We used this property to investigate whether the BIR3 α1-helix is required for Smac mimetic binding. Cells expressing cIAP1 or its Δα1 BIR3 mutant were exposed to Smac-mimetics for 1 hour. As shown in Fig 2G, the Δα1 BIR3 mutant was totally resistant to Smac mimetic-mediated degradation. Altogether, our results demonstrate the importance of the first BIR3 α-helix of cIAP1 in peptide-binding groove-dependent interaction mechanisms.

### cIAP1 stabilizes E2F1 protein expression and modifies the E2F1 ubiquitination profile

**Fig 3:**
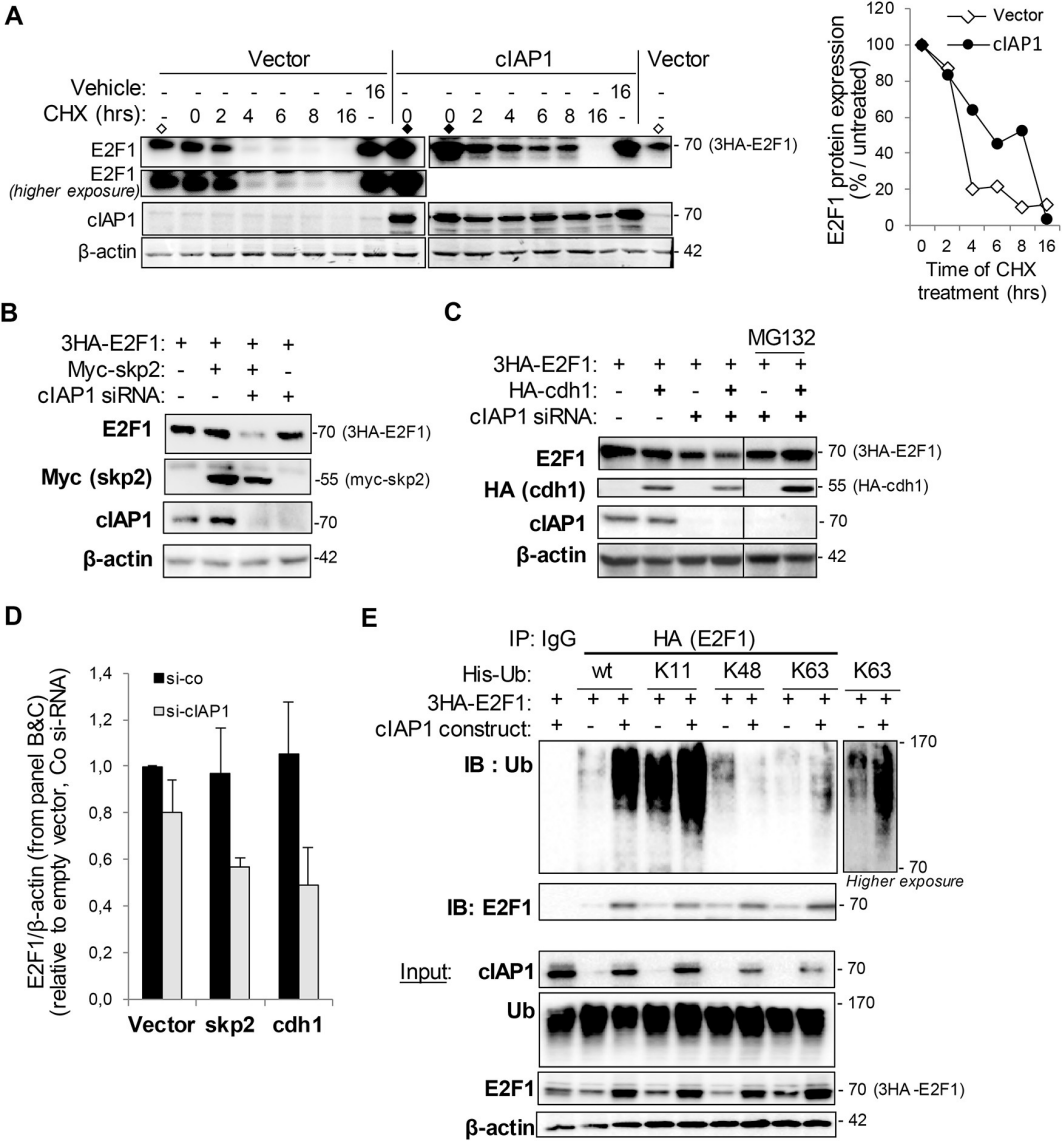
cIAP1 modifies the ubiquitination profile of E2F1. (A) Cycloheximide chase experiment. Cells in which the indicated transgene expression was induced for 48 hrs were treated with cycloheximide 100μg/mL and E2F1 and cIAP1 expression were analyzed by Western blotting. The vector (◊) and cIAP1 (◆) control samples were loaded twice to compare the expression levels. β-actin was used as the loading control. E2F1 expression was quantified using Image J software (right panel). One representative experiment is shown. (B-D) Western blot analysis of 3HA-E2F1, cIAP1 myc-skp2 (B,D) or HA-cdh1 (C,D) in HeLa cells transfected with indicated constructs in the presence of control or cIAP1 siRNA. β-actin was used as the loading control. One representative experiment is shown (B,C). E2F1 and β-actin expression was quantified using Image J software (right panel). Mean ± S.D. of 3 independent experiments are shown (D). (E) cIAP1 modifies the ubiquitination profile of E2F1. HeLa cells were transfected with 3HA-E2F1, His-tagged ubiquitin wt or ubiquitin mutants containing only the K11, K48 or K63 constructs, in the presence of empty or cIAP1-encoding plasmids. E2F1 immunoprecipitated using anti-HA antibody and ubiquitinated was revealed using anti-poly-ubiquitin chain antibody (FK2). The expression of the constructs was checked by Western blot (lower panel).

Fig 2E shows that over-expression of cIAP1 increases the expression of E2F1. This suggests that cIAP1 could stabilize E2F1 protein expression. Accordingly, the expression of cIAP1 increased the half-life of E2F1 in a CHX chase experiment (Fig 3A). We hypothesized that cIAP1 could interfere with the degradation of E2F1. E2F1 is targeted for degradation mediated by the ubiquitin-proteasome system in S and G2/M phases of the cell cycle, specifically by SCF^skp2^ (Skp2 (S phase kinase binding protein 2)-CDC53 (Cullin)-Fbox) and APC/C^cd1^ (anaphase-promoting complex/cyclosome) E3-ubiquitin ligase complexes. We did not observe a decrease in E2F1 protein expression in skp2 or cdh1 overexpressing cells (Fig 3B–3D). However, an siRNA-mediated downregulation of cIAP1 sensitized E2F1 to Skp2 or Cdh1-mediated degradation (Fig 3B–3D). This effect is not observed when the proteasome is inhibited by treating cells with MG132 (Fig 3C). We also observed a moderate decrease in E2F1 protein expression in cIAP1-silencing cells (Fig 3B–3D) confirming that cIAP1 can stabilize E2F1. cIAP1 increased the expression of a poly-ubiquitinated form of E2F1 (Fig 2E). E2F1 has been shown to be modified by several types of ubiquitin chains, with variable consequences [33]. We used His-tagged derivatives of ubiquitin in which all lysines except K11, K48, or K63 had been changed into arginine residues (Fig 3E) to characterize ubiquitination. The ubiquitination profile of E2F1 in HeLa cells was analyzed by immunoprecipitating E2F1 and probing of ubiquitin. We detected a very high level of K11-ubiquitinated E2F1, less of a K48-ubiquitined form and a very low basal amount of the K63-ubiquitinated form of E2F1 (Fig 3E). Overexpression of cIAP1 increased the content of E2F1 modified at K11- and K63-linked ubiquitin chains, but it decreased the proportion of K48-ubiquitinated E2F1 (Fig 3E).

### cIAP1 is required for chromatin anchorage of E2F1

**Fig 4:**
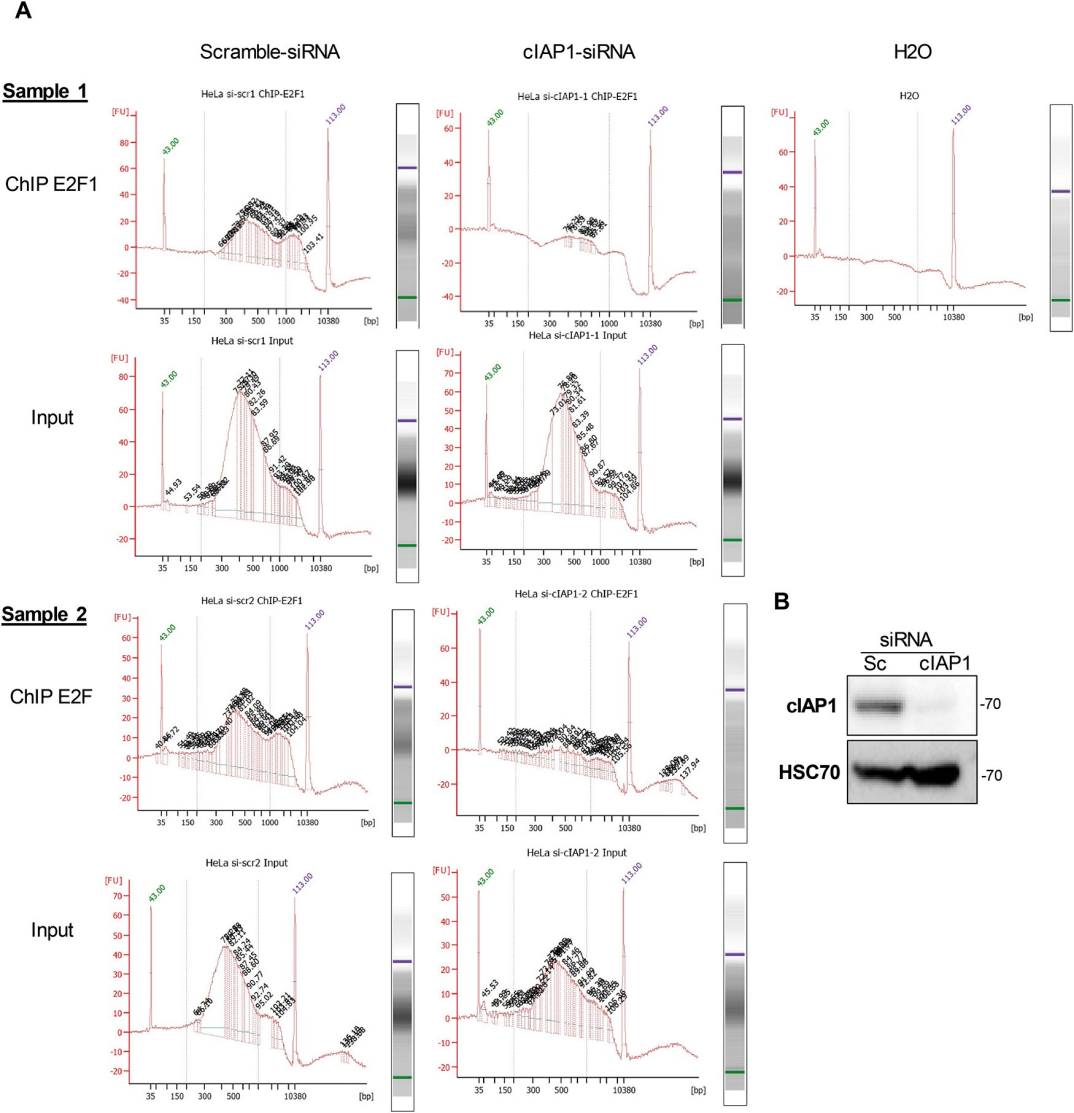
cIAP1 is required for the recruitment of E2F1 onto chromatin. ChIP-seq analysis of the recruitment of E2F1 onto chromatin in HeLa cells transfected with scrambled (Sc) or cIAP1-siRNA. Enriched DNA from ChIP and Input DNA fragments were end-repaired, extended with an ‘A’ base on the 3′end, ligated with indexed paired-end adaptors (NEXTflex, Bio Scientific) using the Bravo Platform (Agilent), size-selected after 4 cycles of PCR with AMPure XP beads (Beckman Coulter) and amplified by PCR for 10 more cycles (A). The efficiency of siRNA was checked by Western Blot analysis (B).

We then attempted to determine the relevance of cIAP1 to the capacity of E2F1 to bind specific target promoters by a Chip-seq analysis in HeLa cells (Fig 4A). Unexpectedly, silencing of cIAP1 (Fig 4B) drastically compromised the global enrichment of chromatin after E2F1 ChIP in two sets of experiments, while the input DNA fragments were amplified in an equivalent manner in both scrambled control and cIAP1 si-RNA samples (Fig 4A). These results suggest that cIAP1 is required for E2F1 binding to DNA in an unspecific manner.

### BIRC2 is an E2F1 target gene

**Fig 5:**
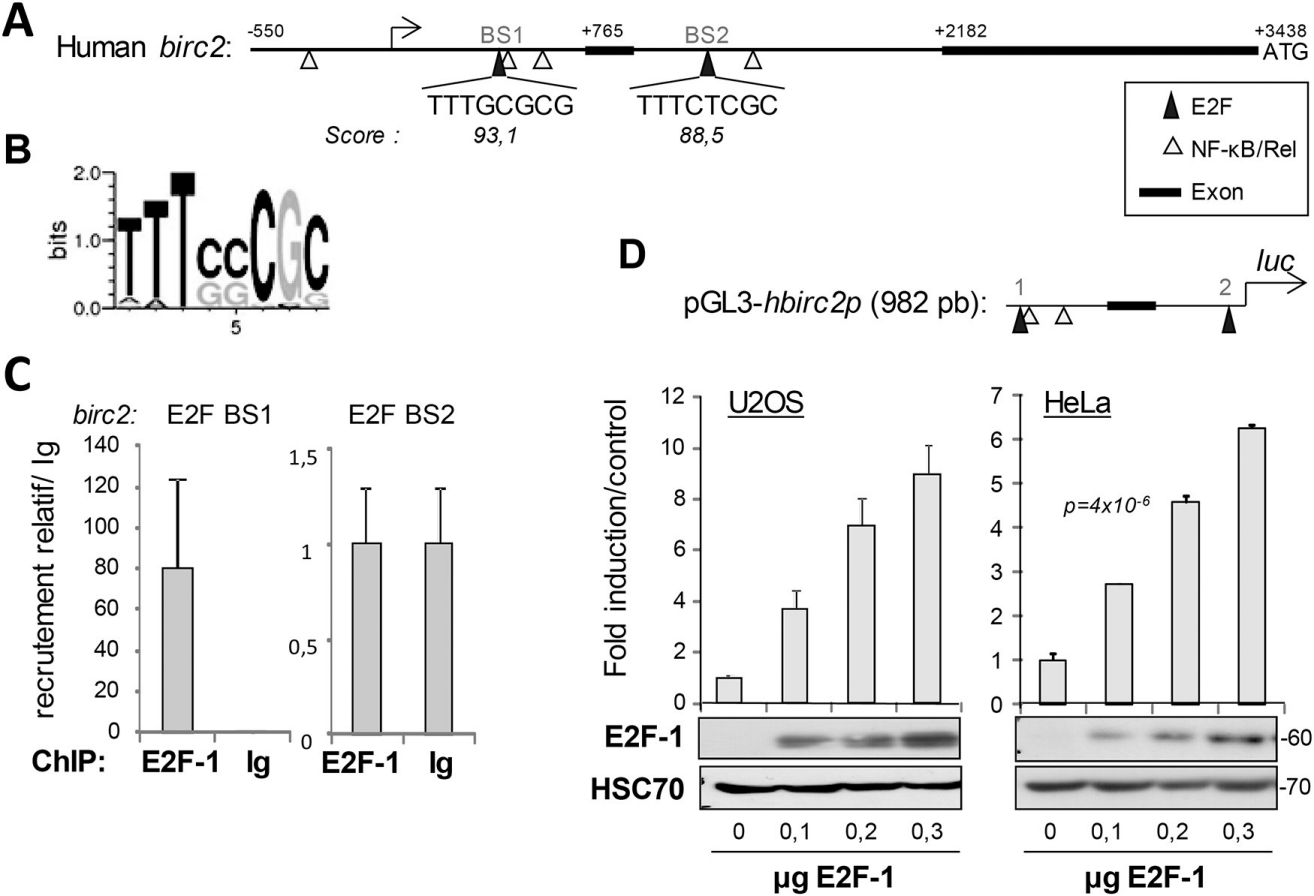
cIAP1-encoding *birc2* is an E2F1 target gene. (A) Representation of the birc2 promoter region. Exons 1 and 2, NF-kB/Rel binding site and putative E2F binding sites (BS1 and BS2) are indicated. (B) Sequence logo representation of the frequency of amino acids in the E2F BS (from the JASPAR CORE database). (C) Chromatin immunoprecipitation experiments were performed using an anti–E2F1 or an irrelevant antibody (Ig). The levels of the E2F1-associated genomic DNA region that encompasses putative E2F binding sites BS1 and BS2 were quantified by qPCR. Results were normalized to input and recruitment with irrelevant antibody, and were expressed as relative recruitment compared to cells transfected with empty vector. (D) Luciferase gene experiments performed in HeLa cells transfected with the *hbirc2* promoter-Firefly luciferase reporter plasmid (upper panel) and increasing concentrations of pCMV-E2F1 constructs. Luciferase activity was normalized to β-galactosidase activity. Results are expressed as a % of the activity measured with wt E2F1. The expression of the constructs was checked by Western blot analysis (lower panel). HSC70 was used as the loading control.

Considering the importance of cIAP1 in E2F1 activity, we examined whether the expression of its transcript could be controlled by E2F1, as was the case for most E2F regulators. We mapped the cIAP1-encoding *BIRC2* promoter sequence for the presence of potential E2F1-binding elements using TFSEARCH software (Searching Transcription Factor Binding Sites (version 1.3) http://www.cbrc.jp/research/db/TFSEARCH.html). We scanned a genomic sequence upstream ATG codon, starting 550 nucleotides upstream of the first transcription start site that had been determined by Young *et al*. [34]. The DNA sequence encompasses the first exon and 4 NF-κB binding sites. We identified two putative E2F binding sites designated as E2F BS1 and E2F BS2 (Fig 5A and 5B). ChIP analysis demonstrated that E2F1 was recruited to the E2F BS1 located between the transcription start site and the first exon (Fig 5C). To determine whether E2F1 can directly activate *birc2* promoters, the *birc2* promoter region containing E2F BSs were cloned into a pGL Luciferase Reporter Vector (Fig 5D) and co-transfected with an E2F1-encoding vector. E2F1 significantly stimulated the *birc2* promoter in a dose dependent manner in HeLa and U-2 0S cells (Fig 5D). We then studied whether Birc2 expression could vary as the cell cycle progressed, as was the case for most E2F1 target genes. Cells were synchronized by a thymidine double block and the birc2 transcript was quantified just after this as well as at 4, 6 and 10 hours after block release. Similarly as for cyclin E, the cIAP1 transcript reached a peak of expression in the G1 phase of the cell cycle (S2 Fig).

## Discussion

The mechanisms of interaction between IAPs and caspases or IAP antagonists have been extensively studied leading to the design of IAP antagonist compounds [35]. Most IAP partners, including Smac, contact a surface hydrophobic groove located in the BIR2 and/or BIR3 domains [36, 37]. We have shown here that E2F1 mostly interacts with BIR3 and uses this interacting pocket, as demonstrated by competition experiments using a Smac-N7-derived peptide or the Smac-mimetic BV6. The peptide-binding pocket generally anchors a specific IBM that has to be exposed at the extreme N-terminus of the protein [6]. Masking the N-terminal extremity of E2F1 does not compromise its interaction with cIAP1, demonstrating the presence of an unconventional interacting motif. Target-assisted iterative screening of peptides able to bind the BIR3 of cIAP1 suggested the existence of unconventional IBM with a consensus NH2-SR(V/P)W sequence [15]. The requirement for exposure of its N-terminus has not been clearly demonstrated. Using truncated mutants of E2F1, we previously demonstrated that the cIAP1 interaction involved an amino-acid fragment covering residues 89–191 that included the DNA binding domain [28]. However, we did not detect either conventional or unconventional IBM-like sequences, and further experiments will be required to identify the E2F1 motif involved in cIAP1 binding. Other BIR3 binding proteins, without IBM motifs, have been described. The Copper Metabolism MURR1 or COMM domain-containing 1 (COMMD1) binds the BIR3 domain of XIAP and cIAP2 [38] through a leucine repeat interface within the COMM domain of COMMD1 [39] and ARTS specifically binds to XIAP-BIR3 [16] via its nine C-terminal residues [40]. Interestingly, the authors highlight the importance of a short amino-acid sequence located in the first α-helix of BIR3 for the ARTS–XIAP interaction [16]. The BIR3 α1-helix is critical for the binding of E2F1, Smac or Smac mimetics whether bivalents (i.e. binding of 2 BIRs) such as Birinapant or monovalent (i.e. binding of only one BIR) such as GDC-0152. Thus, even though it does not directly participate in the peptide-binding pocket, this region could have a major structural function. Accordingly, the BIR3-α1 helix contains the highly conserved arginine residue (R272) that is essential for BIR function [41, 42], stabilizing the tertiary fold of BIRs [42, 43].

The E2F1 transcription factor controls the expression of multiple genes, including those required for the G1-S phase cell cycle transition, and those that regulate the genotoxic stress response [44, 45]. Accordingly, its expression and activity are stringently regulated during cell cycle progression and it is rapidly upregulated and activated in response to DNA damage. Among regulatory mechanisms, post-translational modifications are an extremely efficient strategy to quickly modulate the activity or stability of proteins [46]. E2F1 can be modified by a wide variety of post-translational modifications including methylation, acetylation, phosphorylation, neddylation, sumoylation and ubiquitination. These modifications can occur on different amino-acid residues and a given amino-acid can accept different types of modification, which considerably increases the complexity of the regulation mechanisms [46]. When analyzing the ubiquitination profile of E2F1 in HeLa cells in which the proteasome has been neutralized, we observed that a large proportion of E2F1 was modified by K11-linked ubiquitin chains, whereas there was a medium level of K48-ubiquitin chain-conjugated E2F1 and only a very low amount of K63-ubiquitinated E2F1. We cannot rule out the presence of heterogeneous branched ubiquitin chains [47]. The K11 and K48 ubiquitination of cell-cycle regulators (for instance by the anaphase-promoting complex/cyclosome (APC/C)) has been associated with proteasomal degradation [48, 49]. However, the K11-linked ubiquitin chains can also act as non-degradative signals [50]. K63-ubiquitination is considered to be a non-degradative modification involved in protein activation, translocation or in the assembly of signaling protein complexes [47]. Over-expression of cIAP1 dramatically increases the cellular content of E2F1 and modifies the E2F1-ubiquitination profile. It decreases the ratio of K48-ubiquitinated E2F1 and increases the ratio of E2F1-modified by K11- and K63-linked ubiquitin chains. The drop in the proportion of E2F1 modified with K48-linked ubiquitin chains is consistent with the capacity of cIAP1 to increase the stability of E2F1. K63-linked poly-ubiquitination of E2F1 has been associated with the stabilization of E2F1 in S-phase of the cell cycle and in response to DNA damage [29]. Given that (i) cIAP1 increases the proportion of K63-ubiquitinated E2F1 (in this present work), (ii) cIAP1 is required for the stabilization of E2F1 in S-phase of the cell cycle and in response to DNA damage, (iii) cIAP1 can directly induce the poly-ubiquitination of E2F1 [29], cIAP1 is likely to be one of the E3-ubiquitin ligases responsible for the S phase or DNA damage-associated K63 poly-ubiquitination of E2F1. Importantly, we showed that the depletion of cIAP1 considerably alters the capacity of the transcription factor to bind chromatin, suggesting that this modification could be a general mechanism of regulation for the recruitment of E2F1 to target gene promoters. E2F1 stabilization could be a secondary event, consequent to the binding of E2F1 to chromatin, which can modify the accessibility of E2F1 to degradation machineries. Alternatively, E2F1 stabilization could be linked to the capacity of cIAP1 to modulate the activity of other E3-ubiquitin ligase complexes involved in the regulation of E2F1 content during cell cycle progression. The E2F1/Rb pathway is subject to complex processes of cross-regulation that control its temporal activation during cell cycle progression. Thus, E2F1 can regulate the expression of other members of the family, co-factors and regulators forming feedback regulatory loops [51, 52]. For example, E2F1 controls the expression of the SCF-E3 ubiquitin ligase subunit skp2 [53] that induces the ubiquitination and degradation of E2F1 at the end of the S phase of the cell cycle [54]. Similarly cIAP1-encoding *BIRC2* is an E2F1 target gene. These results add a new layer of complexity to the mechanisms of E2F1 regulation. Thus, although cIAP1 is mainly detected in the cytoplasm, particularly in immune cells, the nuclear fraction of cIAP1 observed in proliferating undifferentiated cells and in some cancer cells appears as an important regulator of E2F1 (28). Interestingly, Cao *et al*. demonstrated that a nuclear-expressed mutant of XIAP can also regulate E2F1 [55].

Altogether, our work described the interaction of E2F1 with the hydrophobic pocket of BIR3 by using an atypical interacting motif. We also demonstrated the importance of the BIR3 α1-helix in the peptide-binding groove-dependent interaction. We highlighted the importance of cIAP1 as a cofactor required for the recruitment of E2F to E2F-target genes. The role of cIAP1 as an E2F1 regulator is strengthened by the demonstration that cIAP1-encoding *birc2* is an E2F-target gene, a property shared by most 2F1 regulators. Dysregulation of E2F1 is a frequent event observed in human cancer cells because of the inactivation of the retinoblastoma protein RB1, altered expression of cyclin-dependent kinases or their inhibitors and/or the expression of transforming viral proteins. Alteration of cIAP1 could be a supplementary event that culminates in the dysregulation of the E2F1/Rb axis in cancer. Inversely, since the cIAP1-encoding gene is also an E2F-target gene, dysregulation of E2F1 can explain the exacerbated expression of cIAP1 observed in human tumors.

## Supporting information

S1 Fig (A) IBM sequence of known cIAP1 protein partners. (B) Immunoprecipitation analysis of the interaction of cIAP1 with E2F1 devoid of its 2 first amino acids (MA) and conjugated in the N-ter position with 3-HA tags. The indicated constructs were expressed in HeLa cells. cIAP1 or E2F1 was immunoprecipitated using an anti-cIAP1 or anti-HA antibody or a control Immunoglobulin. The cIAP1-E2F1 interaction was revealed by Western blot analysis.
(TIF)

S2 Fig HeLa cells were synchronized in early S phase by a thymidine double block, and were analyzed 0, 4, 6 and 10 hrs after block release. (A) Cell cycle analysis of the DNA content stained by propidium iodide. (B) Quantitative RT-PCR analysis of *ccne*, and *birc2* mRNAs in synchronized HeLa cells. Results were normalized to cyclophilin mRNA and were expressed relative to empty vector. Results represent mean +/- S.D. of one representative experiment.
(TIF)
