# Supplementary figures and images for: E2F1 binds to the peptide-binding groove within the BIR3 domain of cIAP1 and requires cIAP1 for chromatin binding
Source: PLoS One. 2018 Oct 25;13(10):e0206253. doi: 10.1371/journal.pone.0206253 (PMC6201919; doi:10.1371/journal.pone.0206253)

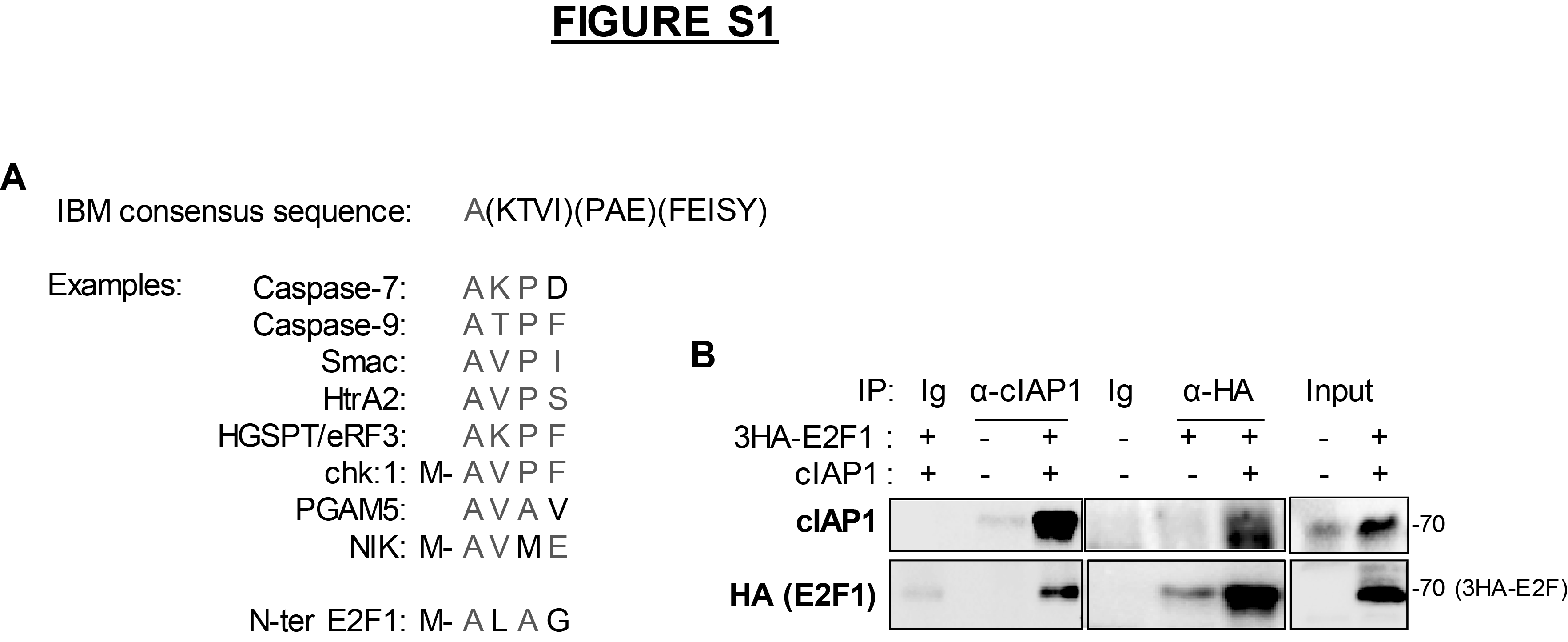

Supplement: S1 Fig — (A) IBM sequence of known cIAP1 protein partners. (B) Immunoprecipitation analysis of the interaction of cIAP1 with E2F1 devoid of its 2 first amino acids (MA) and conjugated in the N-ter position with 3-HA tags. The indicated constructs were expressed in HeLa cells. cIAP1 or E2F1 was immunoprecipitated using an anti-cIAP1 or anti-HA antibody or a control Immunoglobulin. The cIAP1-E2F1 interaction was revealed by Western blot analysis. (TIF) [file pone.0206253.s001.tif]

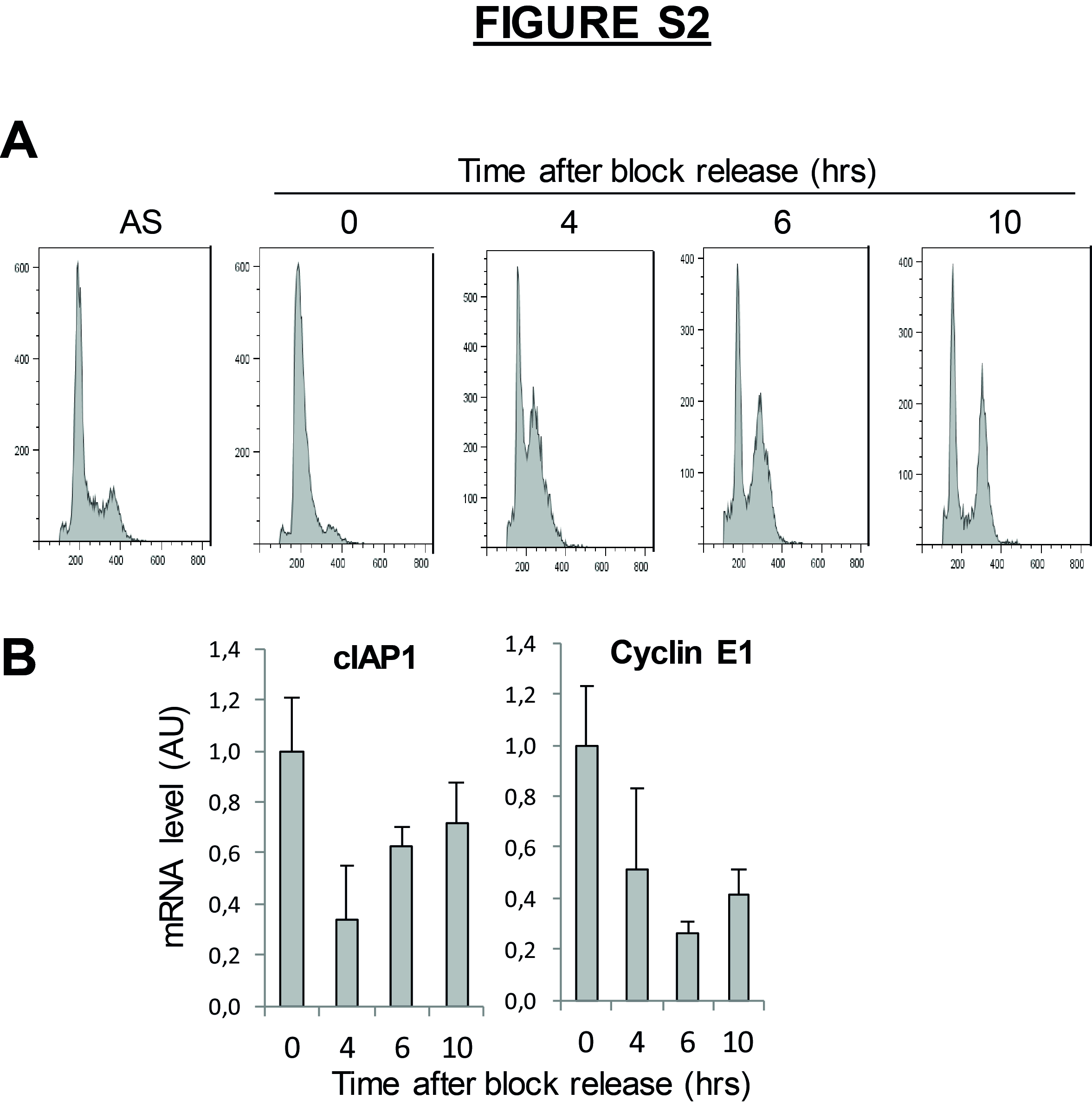

Supplement: S2 Fig — (A) Cell cycle analysis of the DNA content stained by propidium iodide. (B) Quantitative RT-PCR analysis of ccne, and birc2 mRNAs in synchronized HeLa cells. Results were normalized to cyclophilin mRNA and were expressed relative to empty vector. Results represent mean +/- S.D. of one representative experiment. (TIF) [file pone.0206253.s002.tif]
